# Differences in Self-Reported Health in the Osteoarthritis Initiative (OAI) and Third National Health and Nutrition Examination Survey (NHANES-III)

**DOI:** 10.1371/journal.pone.0017345

**Published:** 2011-02-28

**Authors:** William M. Reichmann, Jeffrey N. Katz, Elena Losina

**Affiliations:** 1 Department of Orthopedic Surgery, Brigham and Women's Hospital, Boston, Massachusetts, United States of America; 2 Department of Biostatistics, Boston University School of Public Health, Boston, Massachusetts, United States of America; 3 Division of Rheumatology, Immunology and Allergy, Brigham and Women's Hospital, Boston, Massachusetts, United States of America; 4 Department of Epidemiology, Harvard School of Public Health, Boston, Massachusetts, United States of America; University of Michigan, Canada

## Abstract

**Objective:**

To assess self-reported health status (SRHS) in two cohorts of participants with radiographic knee osteoarthritis (OA) and examine the extent that differences in SRHS are due to study design.

**Method:**

We used data from the Third National Health and Nutritional Examination Survey (NHANES-III; population-based national survey) and the Osteoarthritis Initiative (OAI; prospective cohort study). Inclusion criteria for this analysis were age 60–79 and presence of radiographic knee OA. SRHS, elicited as a five-item domain (excellent, very good, good, fair, poor), was analyzed by dichotomizing the general health status measure as “fair/poor” versus all other states. We estimated the proportion of participants in fair/poor health from each study. Propensity score methodology was used to adjust for the differences in sampling strategies between the two studies.

**Results:**

Thirty-four percent (N = 1,608) of OAI and 29% (N = 756) of NHANES-III participants satisfied inclusion criteria. The proportion in fair/poor health was higher in NHANES-III (28%) than in OAI (5%). After adjusting for the propensity score, the proportion in fair/poor health was four times higher in NHANES-III than in OAI.

**Conclusion:**

SRHS was substantially better in OAI than in NHANES-III. Self-selection bias may contribute to overestimation of SRHS in prospective cohort studies such as OAI.

## Introduction

Knee osteoarthritis (OA) is a prevalent and disabling disease that primarily affects the elderly. Among US adults age 60 or older, knee OA is one of the five leading causes of disability [1] and approximately 12–16% have symptomatic knee OA [2], [3]. Studies have shown that knee OA greatly diminishes health status in the elderly [4], [5].

Self-reported health status (SRHS) is a subjective measure of how one perceives and reports his or her own well-being. SRHS is often measured by asking individuals to rate their health as excellent, very good, good, fair, or poor. This type of self-reported information is considered an important indicator of a person's health status [6]. It has been shown to be a stable measure of one's health and to be associated with the number of physician contacts [7] and mortality [7], [8]. Often SRHS is measured in national surveys to monitor population health and this measure has been used in the United States [8], Canada [6], England [9], and Australia [10].

SRHS in persons with radiographic knee OA could be studied in national population-based studies, such as the Third National Health and Nutritional Examination Survey (NHANES-III), or large longitudinal prospective cohort studies, such as the Osteoarthritis Initiative (OAI). Each study design has its own distinct advantages and disadvantages. An advantage of well-designed population-based studies is that they exhibit good internal and external validity [11]. However, most of these studies are cross-sectional in nature, which makes it challenging to evaluate how SRHS changes over time [12]. An advantage of cohort studies is that most are longitudinal so they are able to evaluate how SRHS changes over time. However, these studies usually rely on volunteer study subjects that may be healthier than the general population rendering the studies vulnerable to selection bias [13]. Selection bias in turn limits the extent that study results can be generalized to the general population.

Propensity score methodology has been used to account for selection bias in non-randomized studies [14]–[17]. This analytic approach assigns each subject a propensity score, defined as the probability of the subject receiving one of the treatments under consideration, as opposed to the other. The propensity score permits investigators to adjust for selection bias due to measured factors, but it still cannot account for selection bias that is due to unobserved factors [18].

Our objective was to report the SRHS from two studies focused on knee OA. We sought to examine the differences in SRHS, and factors explaining these differences, between the two studies. As a methodological aim, we also sought to assess the differences in SRHS due to study design and to evaluate how much of these differences can be remedied by using propensity score methodology. Evaluation of SRHS in these two cohorts will demonstrate the differences in SRHS between a nationally representative sample (NHANES-III) and a volunteer cohort (the OAI). These differences have implications both for interpreting OAI data per se and more generally for the use of volunteer cohorts to understand population level effects.

## Methods

### Ethics statement

The Brigham and Women's Hospital institutional review board approved the study. Since all data is available freely on the web and all data analyses were secondary, we did not obtain written informed consent from the subjects.

### Data sources

#### NHANES-III

NHANES-III is a national population-based survey that was conducted from 1988–1994 by the National Center for Health Statistics of the Center for Disease Control and Prevention. The survey was conducted in two phases. Phase I took place from 1988–1991 and Phase II from 1991–1994. NHANES-III survey data were collected during a face-to-face interview. All participants were then asked to schedule an appointment at a medical examination center where additional data would be collected. Additional details about patient recruitment and selection for the NHANES-III survey has been documented [19].

Radiographs were performed during Phase II for all NHANES-III participants who were 60 years of age or older and could transport themselves to the radiograph table under their own power. The radiographs were performed using a non-weight bearing anteroposterior approach according to NHANES-III protocol [20]. To be included in this analysis, participants had to be between the ages of 60 and 79 with evidence of radiographic knee OA.

#### OAI

OAI is an on-going multi-center, longitudinal, prospective cohort study. Participants were eligible for the study if they were between 45 and 79 years old at entry. Participants were excluded if they had rheumatoid or any inflammatory arthritis, were unlikely to show measurable joint space narrowing, had total knee replacement (TKR) in both knees or planned to have TKR in both knees in the next 3 years, were unable to undergo an MRI, had a positive pregnancy test, were unable to provide a blood sample, used ambulatory aids other than a straight cane, had comorbid conditions that would prevent them from participating in a four-year study, were unlikely to reside in the clinic area for at least three years, were participating in a double-blind randomized control trial at the time, or were unwilling to sign the informed consent. Socio-demographic and clinical data were collected via a computer-based self-administered questionnaire. The radiographs were performed using a posteroanterior fixed-flexion weight-bearing approach according to OAI protocol. For our analysis we further restricted the OAI sample to those between the ages of 60 and 79 and had evidence of radiographic knee OA at their baseline visit.

Data used in the preparation of this article were obtained from the Osteoarthritis Initiative (OAI) database, which is available for public access at http://www.oai.ucsf.edu/. Specific datasets used are version 2.1 of “Enrollees00”, “JointSx00”, “MedHist00”, “PhysExam00”, “SubjectChar00”, and “Biomarkers00”. Additional documentation describing various aspects of the design and methods of the OAI is available on the OAI Online website (http://www.oai.ucsf.edu/).

Differences in study design characteristics are defined in detail in Table 1.

**Table 1:** Comparison of NHANES-III and OAI study characteristics.

	NHANES-III	OAI
Sampling frame	Adults age 18+ residing in the United States	Adults 45–79 residing near one of the four clinical centers that have knee OA or are at risk for developing knee OA
Eligibility criteria (for a knee radiograph)	Age 60+	Age 45–79
	Exclusion: Can not transport themselves onto the x-ray table	Exclusions: Inflammatory arthritis, Advanced knee OA, Bilateral TKR, Unable to have MRI done, Positive pregnancy test, Unable to provide a blood sample, Use of ambulatory aids other than a straight cane for greater than 50% of the time, Comorbid conditions that may interfere with ability to participate, Unlikely to reside in clinic are for at least 3 years, Current participation in a RCT
Data collection period	1991–1994 for radiographs	2004–2006 for baseline data
Data collection methods	All data was collected via face-to-face interview except for radiographic data and BMI	All data was collected via self-administered questionnaire except for radiographic data and BMI
Coding of covariates		
Age	Excluded those 80+; 4 groups: 60–64, 65–69, 70–74, 75–79	Excluded those 45–59; 4 groups: 60–64, 65–69, 70–74, 75–79
Gender	Male versus female	Male versus female
Race	White versus nonwhite	White versus nonwhite
Income	Income was inflated to 2004 dollars using CPI conversion then classified into five groups: <$20,000, $20,000–$34,999, $35,000–$49,999, ≥$50,000, and missing	Five groups: <$20,000, $20,000–$34,999, $35,000–$49,999, ≥$50,000, and missing
Obesity status	Non-obese (body mass index <30) versus obese (body mass index ≥30)	Non-obese (body mass index <30) versus obese (body mass index ≥30)
Comorbidity	0–1 versus 2+ conditions (information on conditions collected in both studies)	0–1 versus 2+ conditions (information on conditions collected in both studies)
Knee pain	Yes versus no; Yes defined as “having knee pain for most days for six weeks or more”	Yes versus no; Yes defined as “having knee pain most days for the past 30 days”
Radiographic Severity	K-L grades 2, 3, and 4 computed by trained radiologist	K-L grades 2, 3, and 4 computed OARSI/ATLAS grades for osteophytes and joint space narrowing, which were computed by trained radiologists

### Defining knee OA: Radiographic assessment

Knee radiographs were assessed by trained radiologists in both NHANES-III and OAI. In NHANES-III, K-L grades were computed by the radiologist. In OAI, osteophyte and joint space narrowing scores were computed for each knee by trained radiologists based on OARSI Atlas grades. From these scores we then computed K-L grades for each knee based on the algorithm provided on the OAI website [21]. For our analysis we used the greater of the right and left K-L grades, and we defined radiographic knee OA in the tibiofemoral joint as having a K-L grade of at least 2.

### Outcome: Self-reported health status

SRHS was elicited in both NHANES-III and OAI by asking the participant to rate their overall health as excellent, very good, good, fair, or poor. As has been done in previous studies, SRHS was analyzed as a dichotomous variable (excellent, very good, or good *versus* fair or poor) [4], [22]–[25].

### Potential correlates of self-reported health status ascertained in both studies

#### Sociodemographic characteristics

We hypothesized that age, gender, race, and income are possible correlates or confounders of SRHS. NHANES-III and OAI had different age criteria to be included in their studies although there was significant overlap. NHANES-III performed knee radiographs on participants age 60 and older, while OAI included participants between the ages of 45 and 79. To best take advantage of these age criteria, we included participants who were between the ages of 60 and 79. Age was classified into four categories; 60–64, 65–69, 70–74, and 75–79. We classified participants in both studies as being of white or nonwhite race. We inflated participant's income in NHANES-III from 1994 dollars to 2004 dollars using the CPI conversion index calculator provided by the US Bureau of Labor Statistics website so that a participant's income in NHANES-III would be comparable to a participant's income from OAI [26]. We then classified income into five categories; <$20,000, $20,000–$34,999, $35,000–$49,999, ≥$50,000, and missing.

#### Comorbidity

We identified several closed-ended questions concerning the presence of medical problems at the time of the survey or in the past in both studies. A comorbidity index was computed by counting the total number of self-reported medical problems. These included asthma, chronic bronchitis or emphysema, congestive heart failure, myocardial infarction, stroke, diabetes, cancer (including skin cancer), fractures of the hip or spine, gout, and back pain most days for at least one month. We then dichotomized the total number of comorbidities as those having 0-1 comorbidities versus 2 or more. Obesity status was considered as a separate factor with obesity defined as body mass index (BMI) greater than or equal to 30 [27].

#### Knee Pain

We defined NHANES-III participants as having knee pain if they answered ‘Yes’ to having knee pain for most days for six weeks or more. OAI participants were considered as having knee pain if they answered ‘Yes’ to having knee pain most days for the past 30 days.

### Statistical analysis

Demographic and clinical features of the two samples are compared using percentages and are displayed in Table 1. We calculated the proportion of participants in fair/poor health along with 95% confidence intervals for both cohorts stratified by the previously described correlates. To account for the over-sampling of minorities in NHANES-III, we applied the appropriate sampling weights for this unadjusted analysis. There were no sampling design features that require the use of sampling weights in the analyses involving OAI participants.

**Figure 1:**
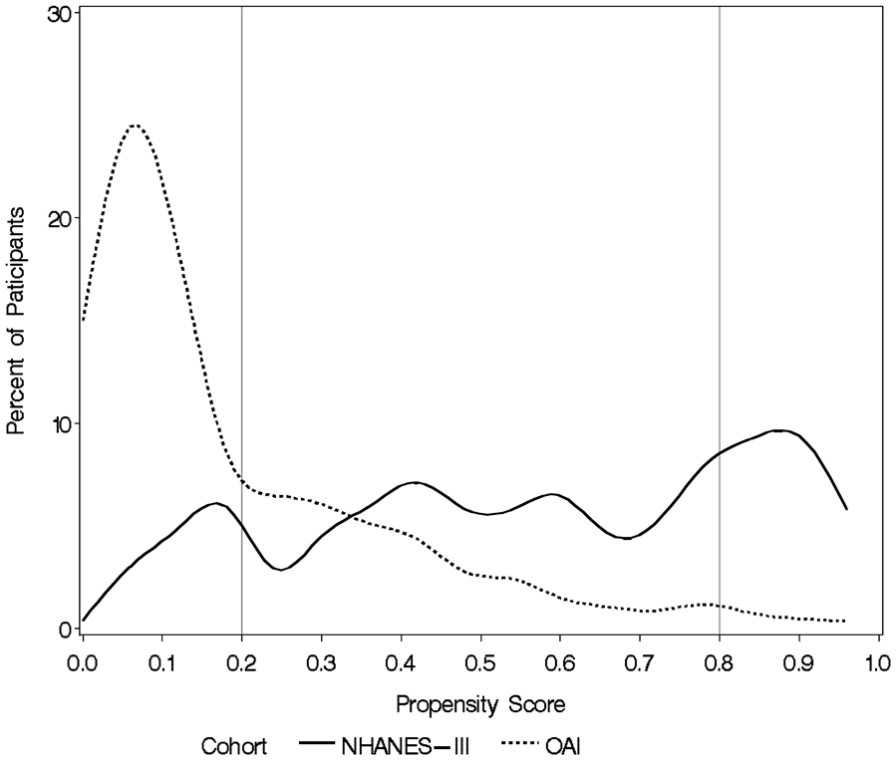
Distribution of propensity scores, defined as the probability of being selected into the NHANES-III cohort, by study (NHANES-III, OAI). The propensity score (x-axis) is defined as the probability of being selected into the NHANES-III cohort. The percentage of individuals with that propensity score (estimated using Kernel density estimation) in each cohort is shown on the y-axis. The solid line represents NHANES-III, while the dashed line represents OAI.

To evaluate the effect of study design on SRHS we conducted five analyses. The first analysis compared SRHS between the two cohorts without adjusting for any other factors using the whole sample. The second analysis compared SRHS between the cohorts by adjusting for the covariates we have previously mentioned using the whole sample. Analyses three through five involve constructing a propensity score model, which estimates the probability of being in the NHANES-III cohort as opposed to the OAI cohort [14]. This was done by performing multivariable logistic regression with study (NHANES-III or OAI) as the dichotomous outcome and age, gender, race, obesity status, comorbidity, K-L grade, knee pain, and income as covariates. Since the propensity score model is evaluating the probability of being selected into NHANES-III given a set of covariate values, we did not apply sample weights to this model. To evaluate the performance of our propensity score model we first trimmed the sample to those with propensity scores between 0.2 and 0.8 because that is the region where there was substantial overlap between the two groups (Figure 1). We evaluated how well the propensity score model was able to balance the covariate distributions between the cohorts by regressing the interaction between cohort status and quintile of the propensity score on each covariate using generalized logistic regression. Statistically significant interactions indicate that given the quintile of the propensity score, the cohorts differ for that particular covariate [14]. In analysis three we compared SRHS between the two cohorts without adjusting for any factors in those who had a propensity score between 0.2 and 0.8 (i.e. those included in the trimmed sample). In analysis four we used multivariable logistic regression to analyze the difference in SRHS between the cohorts while adjusting for covariates in the trimmed sample. In analysis five we used multivariable logistic regression to analyze the difference in SRHS between the cohorts while adjusting for the propensity score in the trimmed sample.

In our models that adjusted for the propensity score or for other covariates we created dummy variables for missing race, obesity status, and income to minimize the number of observations that needed to be removed for missing data.

## Results

### Sample

Two thousand five hundred eighty-six participants completed the NHANES-III household questionnaire and had the physical examination. Of the 2,586 participants, 2,412 (93.3%) had a K-L grade on at least one knee. Of these 2,412 participants, 756 (31.3%) had radiographic knee OA (K-L 2+) and were between the ages of 60 and 79. The OAI cohort consists of 4,796 participants, 4,491 (93.6%) of whom have a K-L grade on at least one knee. Of these, 1,608 (35.8%) had radiographic knee OA and were between the ages of 60 and 79.

The participants in NHANES-III and OAI differed in many respects. NHANES-III participants were likely to have more comorbidities (25% with 2+ comorbidities versus 8% in OAI). OAI participants were more likely to have more severe disease and knee pain than NHANES-III participants. Twenty-four percent of OAI participants had a K-L grade of 4 and 46% had knee pain. In NHANES-III, 6% were K-L 4 and 34% had knee pain. Distributions of age, gender, race, obesity, and income were similar between the two cohorts (Table 2).

**Table 2:** Demographic and clinical features of NHANES-III and OAI participants between the ages of 60 and 79 with radiographic knee OA.

	NHANES-III (N = 756)N (%*)	OAI (N = 1,608)N (%)
Age		
60–64	181 (24.6%)	448 (27.9%)
65–69	200 (28.7%)	437 (27.2%)
70–74	216 (26.2%)	434 (27.0%)
75–79	159 (20.6%)	289 (18.0%)
Gender		
Female	463 (62.5%)	1,003 (62.4%)
Male	293 (37.5%)	605 (37.6%)
Race		
White	353 (78.4%)	1,304 (81.1%)
Non-white	403 (21.6%)	287 (17.8%)
Missing	0 (0.0%)	17 (1.1%)
Comorbidities		
0–1	556 (74.5%)	1,485 (92.4%)
2+	200 (25.5%)	123 (7.6%)
Obesity Status		
Normal	157 (21.9%)	319 (19.8%)
Overweight	318 (44.1%)	663 (41.2%)
Obese	159 (23.1%)	455 (28.3%)
Morbidly obese	70 (7.7%)	170 (10.6%)
Missing	52 (3.1%)	1 (0.1%)
K-L Grade		
2	534 (72.2%)	458 (28.5%)
3	164 (21.6%)	762 (47.4%)
4	58 (6.2%)	388 (24.1%)
Knee Pain		
No	478 (65.9%)	871 (54.2%)
Yes	278 (34.1%)	736 (45.8%)
Missing	0 (0.0%)	1 (0.1%)
Income		
$50,000+	96 (19.7%)	207 (12.9%)
$35,000–$49,999	68 (11.9%)	527 (32.8%)
$20,000–$34,999	203 (31.2%)	483 (30.0%)
<$20,000	323 (31.0%)	266 (16.5%)
Missing	66 (6.2%)	125 (7.8%)

*Percentages are weighted using the NHANES-III sampling weights.

### Self-reported health status (SRHS)

The prevalence of being in fair/poor health for participants in NHANES-III was 28.3% (95% CI: 23.5, 33.2), while the prevalence of being in fair/poor health for OAI participants was substantially lower (5.2% [95% CI: 4.1, 6.3]). NHANES-III participants maintained a substantially higher prevalence of being in fair/poor health across all subgroups. Participants who were of nonwhite race, had more comorbidities, and reported lower incomes were more likely to be in fair/poor health regardless of the cohort. Additionally, OAI participants who were more obese and reported having knee pain were more likely to be in fair/poor health. In NHANES-III, the proportion in fair/poor health increased with increasing obesity status but the 95% confidence intervals overlapped (Table 3).

**Table 3:** Unadjusted proportion of being in fair or poor health for persons with radiographic knee OA from each study (OAI, NHANES-III).

	NHANES-III (N = 756)Percent* (95% CI)	OAI (N = 1,608)Percent (95% CI)
Age		
60–64	24.7% (13.9, 35.4)	5.8% (3.6, 8.0)
65–69	32.8% (25.7, 39.8)	5.0% (3.0, 7.1)
70–74	24.9% (16.5, 33.4)	4.2% (2.3, 6.1)
75–79	30.9% (22.6, 39.2)	5.9% (3.2, 8.6)
Gender		
Female	32.2% (28.2, 36.2)	5.4% (4.0, 6.8)
Male	21.9% (13.7, 30.1)	4.8% (3.1, 6.5)
Race		
White	25.1% (19.8, 30.5)	3.2% (2.3, 4.2)
Non-white	40.0% (32.0, 48.1)	13.7% (9.7, 17.7)
Missing	N/A**	11.8% (0.0, 27.1%)
Comorbidities		
0–1	22.7% (17.8, 27.6)	4.3% (3.3, 5.4)
2+	44.8% (34.7, 54.9)	15.4% (9.1, 21.8)
Obesity Status		
Normal	19.3% (10.6, 28.0)	2.8% (1.0, 4.7)
Overweight	29.0% (25.7, 32.3)	4.2% (2.7, 5.8)
Obese	29.4% (18.5, 40.2)	4.9% (2.9, 6.9)
Morbidly obese	35.8% (21.7, 49.9)	14.1% (8.9, 19.4)
Missing	56.1% (32.7, 79.6)	N/A***
K-L Grade		
2	27.9% (23.0, 32.7)	4.1% (2.3, 6.0)
3	27.6% (16.9, 38.3)	5.0% (3.6, 6.6)
4	36.7% (18.1, 55.2)	6.7% (4.2, 9.2)
Knee Pain		
No	27.3% (21.8, 32.7)	2.4% (1.4, 3.4)
Yes	30.4% (23.5, 37.4)	8.5% (6.5, 10.5)
Missing	N/A**	N/A***
Income		
$50,000+	8.3% (1.9, 14.8)	1.0% (0.0, 2.3)
$35,000–$49,999	34.8% (17.2, 52.5)	2.1% (0.9, 3.3)
$20,000–$34,999	21.9% (13.4, 30.4)	6.4% (4.2, 8.6)
<$20,000	46.5% (36.8, 56.1)	11.3% (7.5, 15.1)
Missing	21.2% (8.0, 34.3)	7.6% (2.8, 12.3)

*Percentages are weighted using the NHANES-III sampling weights.

**Percentage of persons in fair/poor health was not estimated because there were zero persons with a missing race and missing knee pain in NHANES-III.

***Percentage of persons in fair/poor health was not estimated because there was only one person with a missing obesity status and missing knee pain status in OAI.

### Multivariable analysis and propensity score adjustment

The distribution of propensity scores, where the propensity score is defined as the probability of being selected into the NHANES-III cohort, stratified by cohort is shown in Figure 1. The distribution of propensity scores in OAI is right skewed and concentrated in the 0.0 to 0.2 range, while the distribution is fairly uniform across all values in NHANES-III, indicating substantial differences in populations participating in each study. Within the trimmed sample (defined as a propensity score between 0.2 and 0.8), the propensity score was able to balance the distribution of all the covariates by cohort status with the exception of knee pain (p for interaction  = 0.01).

**Figure 2:**
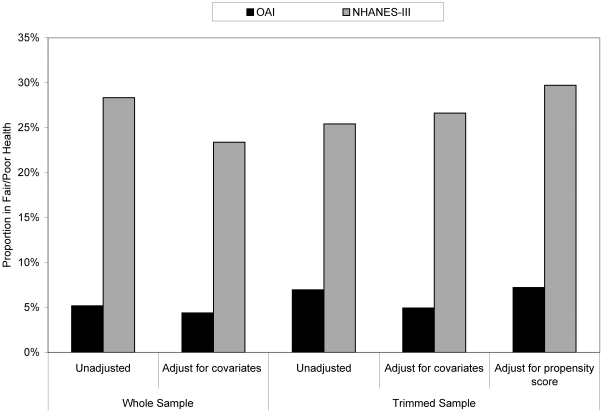
Comparison of the proportion of participants in fair or poor health from in OAI and NHANES-III. On the x-axis is the analysis type and on the y-axis is the percent in fair/poor health. The trimmed sample is defined as participants with propensity scores between 0.2 and 0.8. Dark bars represent OAI, while gray bars represent NHANES-III.

Figure 2 depicts the proportion of participants in fair/poor health stratified by cohort for our five different analyses. When adjusting for all covariates using the whole sample (N = 2,357) the percentage of participants in fair/poor health was 4.4% in OAI and 23.4% in NHANES-III. Trimming the sample (N = 961) based on the propensity score did not substantially account for any of the differences in SRHS regardless of the analysis undertaken. The proportion of participants in fair/poor health in the trimmed sample ranged from 25% to 30% for NHANES-III participants as opposed to 5% to 7% for OAI participants.

## Discussion

We compared SRHS in a national population-based sample (NHANES-III) and a large prospective cohort (OAI) in persons with radiographic knee OA. We analyzed SRHS as a dichotomous outcome using the general health status question. We found that the SRHS in OAI was substantially better (lower prevalence of being fair/poor health) than in NHANES-III. We also found that these differences were not sensitive to any of our adjustment procedures, which included covariate adjustment, and propensity score adjustment with trimming of the sample. In NHANES-III, the prevalence of being in fair/poor health was at least 18% higher regardless of the adjustment procedure.

The implication of this finding is that study design and sampling procedures affect estimates of SRHS. In particular, prospective cohort studies that try to ensure complete follow-up may exclude participants that report a lower health status. Evaluating SRHS in these cohorts may underestimate true population levels and may lead to insufficient power to determine the differences among subgroups since between group differences may be attenuated. This especially holds true for assessing absolute differences and may hold true for assessing relative differences if there is not sufficient variability in the outcome and exposure [11]. In OAI the variability in SRHS is reduced making it difficult to observe clinically and/or statistically significant differences in SRHS between different groups.

Similar to our study, previous estimates of SRHS also appear to be dependent on the study design. A statewide study conducted by Dominick et al. using Pennsylvania's Pharmaceutical Assistance Contract for the Elderly in 1997 found that 55% of OA patients were in fair/poor health, which is much higher than NHANES-III. The mean age of the cohort was 80 years and the mean Charlson Comorbidity Index was 2 [4]. Another statewide study conducted in Missouri by Andresen et al. in 1999 of participants with a mean age of 40 found a 20% prevalence of being in fair/poor health [25]. The Beaver Dam Health Outcomes Study is a community study of participants between the age of 45 and 89. Approximately 12% of participants reported being in fair/poor health, which is higher than the estimate from OAI, but 82% of the participants reported at least one comorbid condition.

Differences in SRHS between OAI and NHANES-III may be due to the two mechanisms of participation. The first mechanism is the exclusion of subjects in OAI. OAI had many more exclusions, which may have precluded the enrollment of participants who have a lower SRHS. The second mechanism is refusal to participate among those who are eligible. Data on participation rates are not made readily available so we can not objectively assess the differences in participation between the two studies. However, the longitudinal nature of the OAI study may be perceived as especially burdensome by eligible participants, especially those in poorer health.

As any analysis utilizing observational studies, our analysis was subject to limitations of such a design. In particular, we were unable to adjust or account for the differences in SRHS observed between the two cohorts that may be due to unmeasured variables. We developed several propensity score models before settling on the one reported in this paper. The propensity score presented in the paper allowed us to maximize the balance covariates between the two studies. Some residual lack of balance across two cohorts with respect to knee pain suggests there is an underlying unmeasured variable or mechanism that may account for some of these differences. These variables could be unmeasured in one or both studies. Another limitation is that the radiographs in NHANES-III and OAI were not performed using the same protocol. NHANES-III used a non-weight bearing approach, while OAI used a more accepted weight bearing method. Because non-weight bearing radiographs were used in NHANES-III, radiographic severity may have been underestimated [28]. While this would affect estimates of SRHS within NHANES-III, it is doubtful that the misclassification of radiographic severity would account for the large discrepancy between the two study populations.

OAI collected SRHS and other data via a computer-based self-administered questionnaire, while NHANES-III collected their data via face-to-face interview. While the impact of survey modality on responses has been well documented when studying topics of sensitive nature, such as HIV [29], the topic has not been rigorously studies when evaluating SRHS. One study found that those responding via a self-administered questionnaire were more likely to report decrements in health-related quality of life [30]. However, in our analysis those responding via a self-administered questionnaire (those in OAI) reported a higher SRHS.

Lastly, SRHS was assessed about 10 years earlier in NHANES-III than in OAI. While it is possible that the time difference could account for the difference it in SRHS, it is unlikely. Hayes et al. conducted analysis of NHANES data collected from 2001-2004 and found that 17% were in fair/poor health [23]. While this is in the middle of the estimates found in NHANES-III and OAI, 78% of the sample was below the age of 60. It is likely that if this analysis was restricted to persons over the age of 60 that the prevalence of being in fair/poor health would increase because of the increase in comorbidity.

We found that SRHS in a national sample of persons with radiographic knee OA was substantially worse than those in a sample from a prospective cohort study conducted in four centers, regardless of the adjustment procedure performed. Since SRHS is an outcome of great interest in persons with knee OA, it is important to note the effect that study design has on this subjective outcome. Strict selection criteria in prospective cohort studies to ensure complete follow-up may make it difficult to study SRHS and how it changes over time because the participants are more likely to have a high SRHS and maintain it over the course of the study. Future studies of SRHS in these cohorts should take into account these possible limitations of their sample.
